# Keyword Analysis: Identifying Interdisciplinary Improvement Opportunities for Sepsis Response

**DOI:** 10.1097/pq9.0000000000000633

**Published:** 2023-02-13

**Authors:** Michael Glassman, Jennifer Treseler, Himi Mathur

**Affiliations:** 1 From the Program for Patient Safety and Quality, Boston Children’s Hospital, Boston, Mass.

## Background:

Boston Children’s Hospital has implemented an enterprise-wide sepsis initiative to manage pediatric sepsis, one of the leading causes of mortality in pediatrics.^1^ To sustain and improve early sepsis identification and treatment, ongoing review of our process and outcomes data highlighted system gaps focusing on interdisciplinary communication and engagement at the bedside.

## Objectives:

We conducted a qualitative assessment for 4 inpatient medicine units with nurses, attendings, residents, and NPs to understand pain points for escalation of concerns and communication methods among the caregivers and inform enterprise-level problem-solving.

## Methods:

**Fig. 1.**
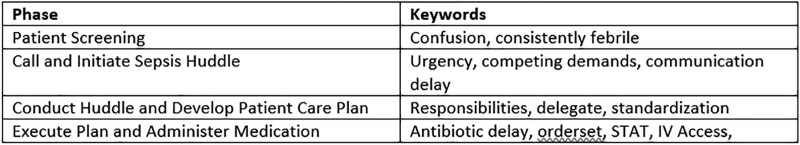
Keywords identified and utilized during data analysis.

We conducted 16 focus group discussions with nurses, candy round interviews with 11 attending physicians, and surveys for 3 nurse practitioners and 47 resident physicians. Over 6 weeks, we collected 406 unique responses across all of our interactions. We then identified basic themes for interdisciplinary improvement in each phase of sepsis recognition and response. We executed a keyword analysis to quantitatively evaluate the responses and highlight opportunities for improvement. Figure 1 illustrates the keywords used in analysis. These specific keywords were identified most frequently throughout our initial thematic analysis. For instance, in our focus group discussions, Orderset and STAT popped up 16 and 20 times, respectively, when evaluating individual responses. We then used Excel’s Wildcard Function to search for multiple keywords at a time from each phase in our sepsis response. This function allowed us to analyze multiple keywords related to each phase of sepsis recognition and response.

## Results:

**Fig. 2.**
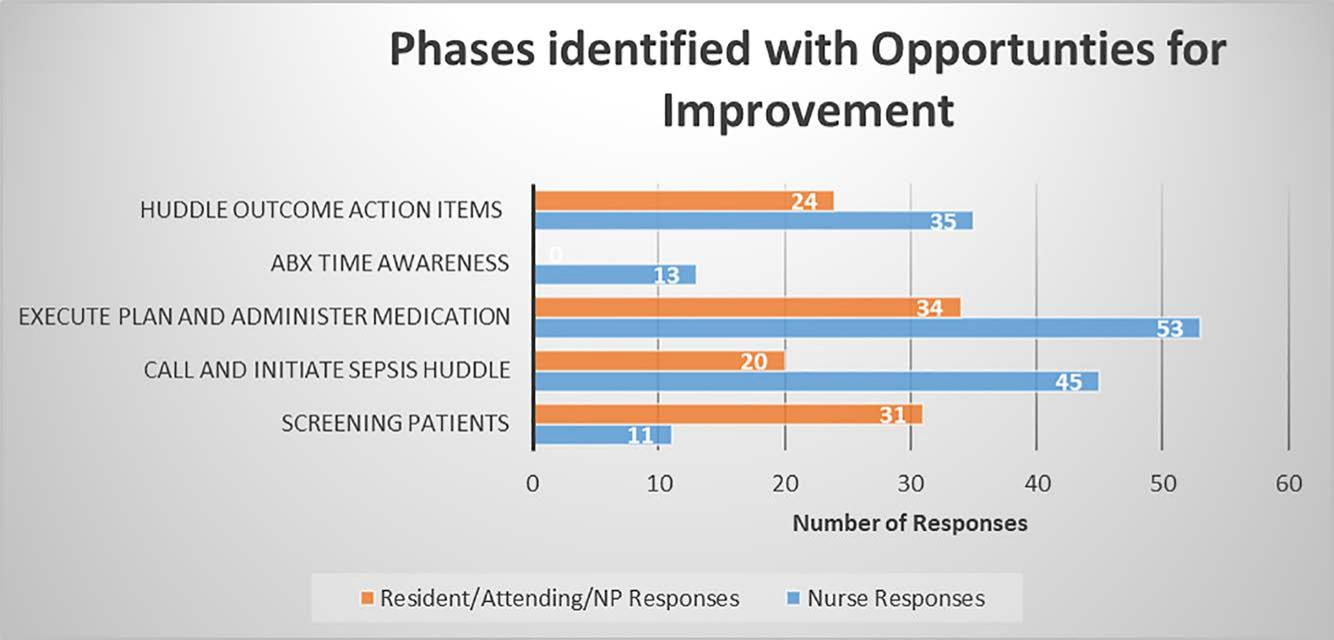
Phases identified with opportunities for improvement. *Note that 116 responses for nurses and 24 responses from Residents, Attendings and NPs fell into miscellaneous categories that were not used in developing interventions.

Figure 2 highlights the improvement themes identified for different phases of sepsis clinical workflow for nurses and prescribers. We identified that the phases of highest uncertainty were during the sepsis huddle, plan of care, and ordering of antibiotics. We noted that in these stages of the care process, our keyword analysis pointed to a limited understanding in the sepsis huddle, lack of communication regarding IV access, and underutilization of our sepsis orderset as areas for improvement. Consequentially, we were able to develop improvement themes based on a Pareto chart developed from the frequency distribution of our keywords.

## Conclusions:

The keyword analysis helped illustrate the interaction of human behavior, technology, and working environments influencing our interdisciplinary workflows surrounding sepsis response. With data as our driver, we partnered with the frontline staff to develop and operationalize our new huddle checklist, IV algorithm triage resource, and sepsis orderset while continuing to monitor both our process and outcome measures. The result was a continuous increase in sepsis bundle utilization for critical sepsis patients with a center line shift from 41% to 70%. One of our biggest drivers of this shift was the centerline shift in sepsis orderset utilization increase from 36% to 61% across all sepsis patients. Finally, in response to our results, we are also working diligently to develop a simulation experience focusing on the huddle process.

## ACKNOWLEDGMENTS

Monica Kleinman, MD, Medical/Surgical Intensive Care, Boston Children’s Hospital; Matthew Eisenberg, MD, Division of Emergency Medicine, Boston Children’s Hospital; Daniel Kelly, MD, Division of Medicine Critical Care, Boston Children’s Hospital; Kate Madden, MD, Medical/Surgical Intensive Care, Boston Children’s Hospital; Teresa Shannon, MSN, RN, CPN; Sue Dixon, RN; and Marlena Smith-Millman, MPH.
